# Anodal Transcranial Direct Current Stimulation Enhances Retention of Visuomotor Stepping Skills in Healthy Adults

**DOI:** 10.3389/fnhum.2020.00251

**Published:** 2020-06-26

**Authors:** Shih-Chiao Tseng, Shuo-Hsiu Chang, Kristine M. Hoerth, Anh-Tu A. Nguyen, Daniel Perales

**Affiliations:** ^1^Neuroscience Laboratory, School of Physical Therapy, Texas Woman’s University, Houston, TX, United States; ^2^Motor Recovery Laboratory, Department of Physical Medicine and Rehabilitation, University of Texas Health Science Center at Houston, Houston, TX, United States

**Keywords:** tDCS, motor learning, rehabilitation, stepping, gait

## Abstract

Transcranial direct current stimulation (tDCS) paired with exercise training can enhance learning and retention of hand tasks; however, there have been few investigations of the effects of tDCS on leg skill improvements. The purpose of this study was to investigate whether tDCS paired with visuomotor step training can promote skill learning and retention. We hypothesized that pairing step training with anodal tDCS would improve skill learning and retention, evidenced by decreased step reaction times (RTs), both immediately (online skill gains) and 30 min after training (offline skill gains). Twenty healthy adults were randomly assigned to one of two groups, in which 20-min anodal or sham tDCS was applied to the lower limb motor cortex and paired with visuomotor step training. Step RTs were determined across three time points: (1) before brain stimulation (baseline); (2) immediately after brain stimulation (P0); and (3) 30 min after brain stimulation (P3). A continuous decline in RT was observed in the anodal tDCS group at both P0 and P3, with a significant decrease in RT at P3; whereas there were no improvements in RT at P0 and P3 in the sham group. These findings do not support our hypothesis that anodal tDCS enhances online learning, as RT was not decreased significantly immediately after stimulation. Nevertheless, the results indicate that anodal tDCS enhances offline learning, as RT was significantly decreased 30 min after stimulation, likely because of tDCS-induced neural modulation of cortical and subcortical excitability, synaptic efficacy, and spinal neuronal activity.

## Introduction

The ability to acquire new motor skills and subsequently retain “learned” motor skills are crucial in our daily lives. Motor skill acquisition refers to improvements in motor performance as a result of practice, whereby movements become automatic and precise (Dayan and Cohen, 2011). Skills such as speaking, writing, and walking are all acquired through repetitive practice/training. To remember and retain such learned skills throughout life, human brains must transform recently learned, fragile motor skills into durable, long-lasting motor memories, through a set of processes referred to as “consolidation,” whereby long-term memories become more stable with time (Krakauer and Shadmehr, 2006; King et al., 2017). Regardless of performance improvements directly resulting from repetitive practice (online skill gains), memory consolidation can result in a continuum of skill improvements between practice sessions, referred to as “offline” skill gains, and evidenced by time-dependent skill improvements that occur within a specific time window after training or following overnight sleep (Borich and Kimberley, 2011; Cantarero et al., 2013; Reis et al., 2015; King et al., 2017).

Transcranial direct current stimulation (tDCS) is a non-invasive, low-intensity brain stimulation technique used to modulate neural excitability and enhance motor performance and learning of hand tasks in humans (Reis et al., 2009, 2015; Reis and Fritsch, 2011; Stagg et al., 2011). The weak tDCS current induces persisting excitability changes in the human motor cortex, lasting up to approximately 90 min after the cessation of stimulation (Nitsche and Paulus, 2000, 2001). These plastic excitability changes are selectively controlled by the polarity, duration, and current strength of the stimulus (Nitsche and Paulus, 2000, 2001; Nitsche et al., 2005). Depending on the polarity of stimulation, tDCS can up- or down-regulate cortical excitability, thereby facilitating or impeding skill performance and learning. Hence, tDCS may be a promising tool to assist motor skill re-training for individuals with neurological disorders after brain injuries, such as stroke. Recent studies suggest that lesions to the primary motor cortex (i.e., M1) have a significant impact on skill re-learning, as a result of decreased cortical excitability post-injury (Dayan and Cohen, 2011; Zimerman et al., 2012). This indicates that the same brain area responsible for controlling motor activity is also involved in memorizing newly learned skills during the early stages of motor learning. The presence of persistent motor control deficits may be attributable to the fact that damage to the brain significantly impacts the ability to acquire motor skills and hence defers the improvement of motor function, including gait.

Visuomotor tasks involve the use of real-time visual feedback to direct a computer cursor toward a visual target while a cursor represents the real-time body motion in space, requiring complex sensorimotor integration through skill practice and learning (Borich and Kimberley, 2011; Dayan and Cohen, 2011; Sarlegna and Sainburg, 2009). In a healthy young population, evidence suggests that visuomotor task training can enhance motor skill performance as it provides real-time visual feedback for the correction of movement trajectory as well as the refinement of motor planning before movement start (Sarlegna and Sainburg, 2009; Shabbott and Sainburg, 2009). In motor learning, skill acquisition (online skill gains) and retention (offline skill gains) are enhanced in healthy adults when anodal tDCS is co-applied with visuomotor hand skill training (Reis et al., 2009, 2015; Reis and Fritsch, 2011; Stagg et al., 2011). Moreover, skill gains after training only occurred when tDCS was applied simultaneously with skill training. Offline skill improvements induced by tDCS are mostly time-dependent, requiring more than 15 min post-stimulation to materialize (Reis et al., 2015). The majority of research studies have examined the effects of tDCS on the recovery of upper limb function in healthy and patient populations (Reis et al., 2009, 2015; Zimerman et al., 2012). A recent study has investigated skill retention of a complex whole-body serial reaction time (RT) task (Mizuguchi et al., 2019); however, there has been a lack of investigations exploring the effects of tDCS on leg skill acquisition and retention (Devanathan and Madhavan, 2016; Seidel and Ragert, 2019). Specific effects of tDCS on visuomotor step training remain unclear. Stepping is an important motor skill for the elderly population, used in response to balance threats (Luchies et al., 1994; Maki and McIlroy, 1997, 2006). Impaired stepping control has been correlated with falls, gait balance deficits, gait dysfunction in the elderly population (Lord and Fitzpatrick, 2001; Cho et al., 2004; Maki and McIlroy, 2006; Melzer et al., 2007; Tisserand et al., 2016).

In this study, we implemented a novel visuomotor stepping task, in which we asked subjects to move their leg forward a pre-determined distance toward a virtual visual target *via* real-time visual feedback of foot trajectory, similar to stepping training used in the clinic to improve walking function. The purpose of this study was to determine whether anodal tDCS, paired with visuomotor step training, can enhance step control in healthy adults. Findings from this study will gude the development of effective multimodal interventions (i.e., brain stimulation with stepping training) to improve walking for people with neurological disorders. We believe that real-time visual feedback will enhance sensory awareness of the distance a leg moves, related to a target location, and ultimately help individuals to regain step control. We hypothesized that this visuomotor step training, in conjunction with anodal tDCS, would improve skill learning and retention, evidenced by decreased step RTs, both immediately (online skill gains) and 30 min after training (offline skill gains).

## Materials and Methods

### Subjects

Twenty self-reported healthy adults [age (mean ± SD), 27.3 ± 4.1 years; 11 females and nine males] participated in the study (Supplementary Table S1). All subjects provided informed consent before participation and the study was approved by Texas Woman’s University Human Subjects Institutional Review Board.

### Experimental Design

**Figure 1:**
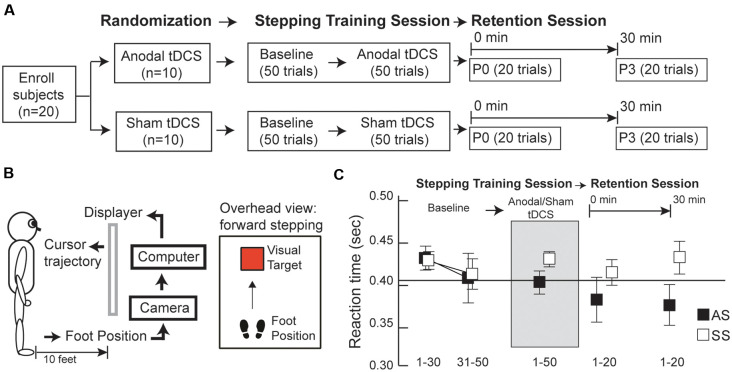
**(A)** Experimental design, **(B)** visuomotor step task setup, **(C)** average reaction times (RTs) for anodal and sham transcranial direct current stimulation (tDCS) groups (AS and SS, respectively) before, during, and after tDCS. **(A)** Subjects were randomly assigned to one of two tDCS groups (anodal or sham tDCS) and underwent two sessions: a training session followed by a retention session. During the training session, subjects first completed 50 stepping trials before tDCS (baseline, BS) and additional 50 stepping trials, combined with either anodal or sham tDCS. In the retention session, 20 stepping trials were conducted at two time-points post-tDCS: 0 and 30 min after tDCS (P0 and P3, respectively). **(B)** Subjects learned to move the foot cursor to a visual target using real-time visual feedback. **(C)** Group average RTs were calculated before, during, and after tDCS. Before tDCS, the average of the last 20 trials at BS was comparable between the anodal and sham groups. After tDCS, anodal the tDCS group exhibited continually decreasing RT values over time, whereas the opposite trend of changes in RT was observed in the sham group. Error bars, ±1 SEM.

The investigation was a randomized-controlled, single-blinded study. After enrollment, participants were randomly assigned to one of two groups: anodal tDCS (i.e., genuine brain stimulation) or sham tDCS (i.e., placebo brain stimulation) group. Participants were blinded from their group assignments and had never previously enrolled in a tDCS study. The study design comprised two testing sessions on the same day: a “step training” session, followed by a “skill retention” session (Figure 1A). During the “step training” session, subjects completed a total of 100 stepping trials. Our pilot research demonstrated that healthy adults would be fully accustomed to this step task within 50 trials, as evidenced by minimal changes in stepping performance. Thus, all subjects first completed 50 step trials before brain stimulation (i.e., baseline, BS), followed by 50 trials with brain stimulation; subjects were seated while the brain stimulation apparatus was set up and run for approximately 5 min, to check the contact quality of the electrodes and assess safety, comfort levels, and tolerance of tDCS. Thereafter, all subjects continued to participate. A 1 min break was included for every block of 10 step trials, to minimize the effects of fatigue throughout the entire “step training” session. In the “skill retention” session, step performance was re-tested at two time-points: (1) 0 min post-tDCS (P0), when 20 step trials were recorded, to quantify immediate effects of brain stimulation on “online learning”; and (2) 30 min post-tDCS (P3), when an additional 20 step trials were conducted, to assess the after-effects of brain stimulation on “offline” learning.

### tDCS Protocols

After completion of the first 50 stepping trials, 20 min of brain stimulation (either sham or anodal tDCS) was delivered through a pair of saline-soaked sponge electrodes (5 cm by 7 cm) using a Soterix 1 × 1 Medical tDCS Low-Intensity Stimulator (Model 1300A, Soterix Medical Inc., New York, NY, USA). Based on the EEG-electrode positions of the international 10/20 system, the medial edge of the anodal or sham electrode was placed lateral to the vertex (Cz) to target the leg area of the primary motor cortex (M1), which controls muscle activations of the stepping leg, and the reference electrode was placed over the supraorbital ridge ipsilateral to the stepping leg (Jeffery et al., 2007; Madhavan and Stinear, 2010). The skin was cleaned before stimulation, to reduce resistance to the electrical current. For **anodal stimulation**, the stimulus intensity was set to 2 mA (current density = 0.057 mA/cm^2^) over a 20-min period. For **sham stimulation**, the direct current was first ramped up to 2 mA, within 30 s at the start of stimulation, immediately followed by a 20-s period when the current continued to ramp down from 2 mA to 0 mA. Subsequently, the current output was decreased to 0 mA over the remainder of the 20-min period. Subjects were informed that it is normal for the perception of brain stimulation (i.e., tingling sensation) to decrease over time, as a result of sensory adaptation to the same stimulation, and were not informed as to whether they were assigned to sham or anodal stimulation during the step test.

### Stepping Task

Subjects were instructed to maintain a normal quiet standing position and were given real-time visual feedback about their leg movements *via* a foot cursor (7 cm by 5 cm) displayed on the wall 10 feet away from the front view (Figure 1B). A reflecting marker was attached to the base of the second toe of the stepping foot (i.e., the preferred, leg for step initiation, SI) to indicate the real-time cursor location on the display and the task was to move the cursor from a starting location to a target. In each trial, the target was presented on the screen at a pre-determined distance, equal to 40% of the individual’s body height (Tseng et al., 2009, 2010), and subjects were instructed to initiate a forward step with the preferred stepping leg, to move the cursor onto a visual target as soon as they saw the target, followed by another forward step made by the other leg to move the whole body from the starting location to the target location.

### Data Collection

A three-dimensional camera system (Flex 13 OptiTrack, NaturalPoint Inc., Corvallis, OR, USA) recorded real-time marker locations during each stepping trial. Customized programming in Visual C++ (Microsoft visual studio, Microsoft Inc., Albuquerque, NM, USA) was used to control real-time cursor motion, as well as the location of the visual target displayed on the wall screen. All data were collected at 100 Hz. Each subject completed the “step training” sessions (including 50 trials before tDCS and 50 trials during tDCS) in approximately 30 min, followed by the “retention” session completed in approximately 40 min (20 trials at 0 min and 20 trials at 30 min post tDCS).

### Data Analysis

A custom Matlab program (MathWorks, Natick, MA, USA) was used for all data processing and analyses. Offline foot position data were filtered using a 2nd-order Butterworth zero phase-lag low-pass filter, with a cut-off frequency of 10 Hz (Tseng et al., 2009). SI was defined as the time when the linear velocity in the forward/backward direction exceeded 1% of its maximal value and the linear velocity continued to increase for more than a second during a forward step. This velocity threshold was determined based on our previous pilot research and coincided with the onset of the lifting of the stepping foot. Step RT was calculated as the time interval between the onset of visual target appearance and the onset of SI in each stepping trial. Average RT values were calculated from the 20 stepping trials across the three time points: (1) BS (average of the last 20 trials); (2) P0; and (3) P3.

To compare the effects of anodal vs. sham tDCS on online and offline skill learning, we calculated the percentage change in RT after, relative to before, tDCS, normalized to the average of the last 20 baselines RTs (Devanathan and Madhavan, 2016). This allowed us to account for differences among individuals, thereby comparing changes in RT due to tDCS (anodal vs. sham). A percentage of 0 indicates no change in the reaction after tDCS. Group means were calculated for each time point (BS, P0, and P3).

To quantify changes in stepping performance before and after tDCS, average movement time (MT) and step accuracy (SA) were calculated across the first 20 trials in BS, the last 20 trials in BS, P0, and P3. Step termination (ST) was defined as the time when the linear velocity in the forward/backward direction fell below 1% of its maximal value and the linear velocity continued to decrease for more than a second during foot landing. MT was determined as the time duration between SI and ST. SA was quantified by the linear distance between the end-point foot position during a forward step and the location of the visual target in the horizontal plane referred to absolute error.

### Statistical Analysis

Statistical comparisons were made using SAS/STAT software (SAS, Cary, NC, USA). A two-way (group × time) mixed-model ANOVA, with repeated measures on one factor (time), was used to test for the effects of time (BS, P0, and P3) and group (anodal vs. sham tDCS). If a significant interaction was present, *post hoc* analyses were performed using Tukey’s Honest Significant Difference test. The level for statistical significance was set at *P* ≤ 0.05.

## Results

**Figure 2:**
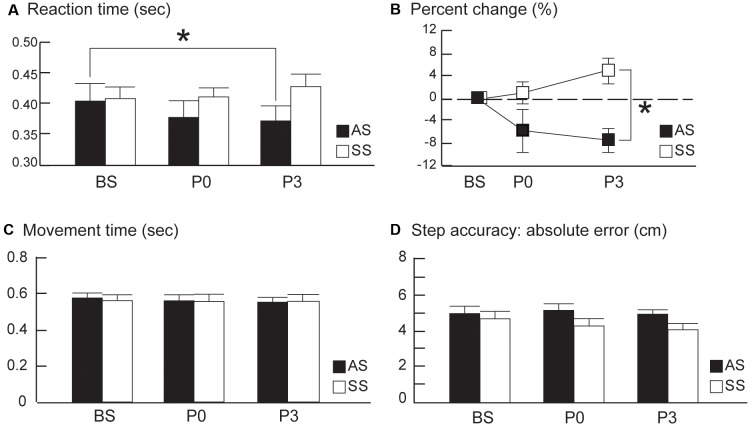
**(A)** Average stepping reaction times (RTs). **(B)** Average percent changes from the baseline RTs, **(C)** average movement time, and **(D)** average step accuracy from pre- to post-tDCS in the anodal and sham tDCS groups (AS and SS, respectively) before (baseline, BS) and 0 and 30 min after tDCS (P0 and P3, respectively). The averages of the last 20 trials at BS were compared to the averages of 20 trials at P0 and P3 between the two groups. Error bars, ±1 SEM. Asterisks (*) indicate significant *post hoc* differences between conditions.

Group averages of RT data for conditions (BS, during tDCS, P0, and P3) are presented in Figure 1C. At baseline, RT averages for the last 20 trials were comparable between the anodal and sham tDCS groups (Supplementary Table S2); indicating all participants were accustomed to the step test before tDCS. Interestingly, RTs post-tDCS showed distinctively different patterns of change in the two groups. After tDCS, the anodal group had decreased RT values at P0 and P3, relative to baseline whereas the sham group showed increased RT at P0 and P3 relative to baseline Comparison of RT values across the three time points (BS, P0, and P3) revealed a significant group × time interaction effect (*F*_(2,36)_ = 6.38, *P* = 0.009, *η*^2^ = 0.02; Figure 2A); however, there were no primary effects of group (*F*_(1,18)_ = 1.04, *P* = 0.32, *η*^2^ = 0.05) or time (*F*_(2,36)_ = 1.00, *P* = 0.39, *η*^2^ = 0.005). In the anodal tDCS group, RT values declined continuously post-stimulation; however, RT at P0 did not differ significantly from baseline (*post hoc* *P* = 0.28); however, at P3 the RT value was significantly lower than baseline (*post hoc* *P* = 0.047). In the sham tDCS group, there were no significant differences in RT from baseline at either P0 or P3 (*post hoc* *P* = 1.0 and *P* = 0.4, respectively).

After normalization to baseline RT, the percent change in RT in the anodal tDCS group exhibited a continuous decline of up to 30 min after brain stimulation, whereas the sham tDCS group showed no decrease in RT after stimulation (Figure 2B). In the anodal tDCS group, RT values were decreased by 5.74% and 7.41% at P0 and P3 from the pre-stimulation baseline value. In contrast, RTs in the sham tDCS group increased by 0.98% and 4.86% at P0 and P3 from the pre-stimulation baseline value. Comparison of percent change in RT across the three time points (BS, P0, and P3) revealed a significant group × time interaction effect (*F*_(2,36)_ = 4.92, *P* = 0.013, *η*^2^ = 0.13) and primary effect of group (*F*_(1,18)_ = 10.09, *P* = 0.005, *η*^2^ = 0.20). However, there was no primary effect of time (*F*_(2,36)_ = 0.74, *P* = 0.48, *η*^2^ = 0.02).The percentage change in RT at P0 did not differ significantly between the anodal and sham groups (*post hoc* *P* = 0.24); however, the difference between the groups became significant at P3 (*post hoc*, *P* = 0.002). Taken together, these findings suggest that anodal tDCS does not cause an immediate reduction in RT value; however, it does reduce RT at 30 min post-stimulation. The lower percentage change in RT during the retention phase (i.e., P3) is likely attributable to the persistent effects of anodal tDCS.

MT, RT, and SA were significantly improved from the first 20 baseline trials to the last 20 baseline trials of BS for both anodal and sham groups, indicating a training effect for this stepping task before tDCS (Supplementary Table S2). After tDCS, MT and SA remained at the similar levels at P0 and P3 relative to the mean of last 20 baseline trials (Figures 2C,D) for both groups, suggesting stepping movements became automatic after baseline training. For MT, there were no effects of group (*F*_(1,18)_ = 0.01, *P* = 0.91, *η*^2^ = 0.0006) or time (*F*_(2,36)_ = 0.63, *P* = 0.54, *η*^2^ = 0.003); nor was interaction effect of group by time (*F*_(2,36)_ = 0.29, *P* = 0.75, *η*^2^ = 0.002). For SA, there were no effects of group *F*_(1,18)_ = 2.27, *P* = 0.15, *η*^2^ = 0.08), time *F*_(2,36)_ = 0.95, *P* = 0.39, *η*^2^ = 0.01), or interaction effect *F*_(2,36)_ = 1.01, *P* = 0.38, *η*^2^ = 0.01).

## Discussion

To our knowledge, this study is the first to demonstrate that the co-application of anodal tDCS and visuomotor step training significantly reduces stepping RT in healthy adults. In this study, we investigated the effects of anodal tDCS on RTs during learning of a visual stepping task in healthy adults. The results showed that stepping RT was significantly reduced at 30 min post-anodal tDCS, while there was no significant decrease at 0 min post-stimulation. These findings suggest that anodal tDCS over the M1 leg area, paired with the learning of a visual stepping task, was effective in promoting skill retention in healthy adults. Future studies should determine the long-term effects of anodal tDCS combined with visuomotor step training on cortical and spinal excitability, to develop therapeutic strategies to enhance the health of people with neurological disorders.

### Significant After-Effect of Anodal tDCS on Stepping Reaction

The significant decrease in stepping RT detected 30 min post-stimulation can likely be attributed to an after-effect associated with anodal tDCS. Previous studies have demonstrated that increased M1 excitability persists, even after cessation of stimulation, referred to as a long-lasting “after-effect” induced by anodal tDCS (Nitsche and Paulus, 2000, 2001). Although the exact neurophysiological mechanisms underlying this effect are incompletely understood, evidence supports that tDCS can modulate neural excitability of the cerebral cortex in rats and humans by changing the polarity of the resting membrane potential in the nervous system (Bindman et al., 1962, 1964; Nitsche and Paulus, 2000, 2001; Nitsche et al., 2005). Specifically, anodal tDCS increases the neural excitability of the stimulation area, whereas cathodal tDCS decreases the neural excitability of the stimulation area. The mechanism underlying the associated polarity-dependent modulations is that anodal tDCS shifts the resting membrane potential closer to the depolarization threshold, thereby increasing neural excitability and firing rate (Bindman et al., 1962, 1964). In contrast, cathodal tDCS shifts the resting membrane potential further away from the depolarization threshold, resulting in hyperpolarization and a decrease in neural excitability and firing rate. Furthermore, in healthy adults, Nitsche and Paulus (2001) first demonstrated that a single session of anodal tDCS over the hand area of the M1 can produce a persistent after-effect, which increased neural excitation to up to 150% of its baseline value; this excitatory effect lasted for approximately 90 min after the end of stimulation. Such after-effects are partially controlled by modulation of N-methyl-d-aspartate (NMDA) receptor efficiency (Liebetanz et al., 2002; Nitsche et al., 2005). Similar to the findings from studies of the upper extremities mentioned above, Jeffery et al. (2007) was the first group to investigate the effects of anodal tDCS on neural excitability of the M1 leg area, which is located at a deeper position, relative to the M1 hand area in humans. These researchers showed that anodal tDCS (2 mA, 10 min) was effective in increasing motor evoked potentials (MEPs) in the M1 leg area of up to 140% of its baseline value 30 min post-stimulation; this excitatory effect continued for approximately 60 min after cessation of stimulation. Data from this study indicate that a 20 min anodal tDCS over the M1 leg area produces similar long-lasting after-effect of increased cortical neural excitability, which is responsible for the observed decrease in stepping RT 30 min post-stimulation.

### No Immediate Effect of Anodal tDCS on Stepping Reaction

Interestingly, in this study, anodal tDCS did not induce a significant decrease in stepping RT immediately after stimulation; however, a significant decrease in RT was observed at 30 min post-stimulation. This finding may indicate that the changes in cortical excitability induced by anodal tDCS did not reach a maximum immediately after cessation of the stimulation and that the anodal tDCS-induced increase in cortical excitability required several minutes to elapse to reach its peak (Bindman et al., 1964; Jeffery et al., 2007). Bindman et al. (1964) showed that, in rats, a higher positive current flow passing through the somatosensory cortex can result in the complete abolition of the evoked potentials (referred to as cortical depression); however, the potentials gradually recovered during the next 30 min and reached a peak value around 30 min after stimulation. Notably, the amplitude of evoked potentials was significantly increased once they returned 30 min later, despite the period of depression. It is possible that, in this study, the positive current of 2 mA flowing over the M1 leg area continuously for 20 min may cause temporary cortical depression, leading to the lack of a significant decrease in RT immediately after brain stimulation. Nevertheless, following anodal stimulation, cortical excitability gradually increased and peaked at 30 min post-anodal tDCS, thereby contributing to the observed significant decrease in RT 30 min later. Jeffery et al. (2007) reported no significant increase in MEPs in the M1 leg area, relative to its baseline value, immediately after 10 min of anodal tDCS over the M1 leg area; however, MEPs continued to increase over the subsequent 60 min, becoming significantly different from the baseline value from 10 min post-stimulation. Behaviorally, Devanathan and Madhavan (2016) have reported that healthy young adults showed decreased RT for ankle choice reaction task 5 min after a single anodal tDCS session whereas prolonged RT was observed after a single sham tDCS session.

### Limitations of This Study

It is difficult to ascribe specific neuronal mechanisms to the findings of this study, due to several limitations. Although evidence indicates that anodal tDCS is responsible for long-lasting after-effects of increased cortical and spinal neuronal excitability in humans (Nitsche and Paulus, 2000, 2001; Lang et al., 2005; Nitsche et al., 2005, 2007; Jeffery et al., 2007; Roche et al., 2009, 2011), we did not measure changes in MEPs before and after the 20 min anodal and sham tDCS while learning a visuomotor stepping task. Therefore, we have limited understanding of the extent of changes in cortical excitability induced by this stepping task combined with anodal tDCS during the training session and the after-effects associated with anodal tDCS in the retention session. Future studies should include transcranial magnetic stimulation (TMS) to quantify the changes in MEPs induced by tDCS during learning of a visual stepping task.

Although a considerable effort was devoted to controlling for the variability attributable to the individual participants, we acknowledge that stepping performance can be influenced by various factors, thereby affecting the RT calculation (Li et al., 2015). It is possible that our participants became bored or fatigued after repeatedly performing the same stepping task, which, in turn, influenced their stepping RTs. It is established that anodal tDCS over the M1 area can improve endurance time and negate fatigue effects (Cogiamanian et al., 2007; Devanathan and Madhavan, 2016). Also, the single-blinded randomized-controlled study protocol used in this study may result in biased outcomes because the researchers were likely to be biased in favor of the intervention they were performing. Furthermore, tDCS may induce widespread cortical changes in the adjacent area of M1 including supplementary motor area and alter functional connectivity between the M1 and motor association cortices, due to its low spatial focality, derived from the relatively large stimulation electrode (35 cm^2^) and dispersed electrical field (Lang et al., 2005; Nitsche et al., 2007). The findings from this study may be attributable to the sum of cortical, subcortical, and spinal neural modulations, rather than only changes in M1 leg area excitability (Lang et al., 2005; Nitsche et al., 2007; Roche et al., 2009, 2011; Polanía et al., 2011; Mizuguchi et al., 2019).

### Clinical Implications for Motor Learning and Gait Rehabilitation

This study advances understanding of the aggregate effects of anodal tDCS and step training on a group of healthy subjects. We demonstrate the feasibility of using anodal tDCS as an adjuvant to step training to reduce stepping RTs and enhance skill retention in healthy adults. These findings may have important clinical implications for the geriatric population and individuals with neurological disorders, whose stepping RTs are significantly longer than those of healthy adults, leading to a limited ability to initiate stepping strategies in response to balance threats and increased risk of falling (Luchies et al., 1994; Maki and McIlroy, 1997, 2006; Tseng et al., 2009; Melzer et al., 2007; Peterson et al., 2016). Furthermore, it has been suggested that age-related slowing in volitional stepping is an important indicator of declined mobility, impaired balance, and increased risk of falling in the elderly population (Lord and Fitzpatrick, 2001; Cho et al., 2004; Maki and McIlroy, 2006; Melzer et al., 2007; Tisserand et al., 2016). The current study demonstrates that combining anodal tDCS and visuomotor step training can reduce stepping RT and therefore this approach may mitigate age-related stepping slowness, whether triggered by external perturbations or self-initiated.

In motor learning, skill acquisition (online learning) and retention (offline learning) were enhanced in healthy adults when anodal tDCS was co-applied with visuomotor hand skill training (Reis et al., 2009, 2015; Reis and Fritsch, 2011; Stagg et al., 2011). Behaviorally, tDCS-induced improvements in visuomotor skill, dependent on the passage of time after training, but not on overnight sleep (Reis et al., 2015). Reis et al. (2015) showed that co-application of tDCS and skill training is essential for the promotion of offline skill gains and prevention of skill loss after training; whereas application of tDCS alone after skill training did not lead to the acquisition of any offline gains; however, the majority of learning studies investigated skill gains in the upper extremities (Reis et al., 2009, 2015; Borich and Kimberley, 2011; Reis and Fritsch, 2011; Stagg et al., 2011; Cantarero et al., 2013). More evidence is needed to determine whether the co-application of anodal tDCS and lower extremity motor training can enhance online and offline skill gains. The results of this study are consistent with those of previous studies of visuomotor hand skill learning, which showed that that co-application of tDCS and skill training is essential to promote offline skill gains and prevent skill loss after training (Reis et al., 2015). Although findings from this study are exploratory, they raise the possibility that repetitive use of anodal tDCS combined with lower extremity motor skill training could help to restore walking function in individuals with neurological disorders, including chronic stroke. Future studies are necessary to understand whether regular anodal tDCS and locomotor training may influence the excitatory state of the M1 leg and spinal neuronal networks in healthy adults, as well as individuals with neurological disorders.

## Data Availability Statement

All data generated or analyzed during this study are included in this published article and its Supplementary Material.

## Ethics Statement

The studies involving human participants were reviewed and approved by Texas Woman’s University IRB Houston. The participants provided their written informed consent to participate in this study.

## Author Contributions

S-CT and S-HC contributed to the conception and design of the study. KH, A-TN, and DP conducted the study. S-CT contributed to data analysis and writing the manuscript.

## Conflict of Interest

The authors declare that the research was conducted in the absence of any commercial or financial relationships that could be construed as a potential conflict of interest.
