# Supplementary material for: Anodal Transcranial Direct Current Stimulation Enhances Retention of Visuomotor Stepping Skills in Healthy Adults
Source: Front Hum Neurosci. 2020 Jun 26;14:251. doi: 10.3389/fnhum.2020.00251 (PMC7333563; doi:10.3389/fnhum.2020.00251)
Supplement: Supplementary file 1 [file Table_1.DOCX]

Table 1. Characteristics of study participants (Means ± Standard Deviations)

|  | Anodal tDCS | Sham tDCS |
| --- | --- | --- |
| N | 10 | 10 |
| Age (years) | 26.80 ± 3.46 | 27.70 ± 4.74 |
| Gender | 4F, 6M | 7F, 3M |
| Body Weight (Kg) | 75.50 ± 22.06 | 73.10± 16.55 |
| Body Height (cm) | 171.20 ± 12.36 | 167.50± 9.90 |
| Step distance (cm) | 68.40 ± 4.79 | 66.40 ± 3.84 |

F: female, M: male

Table 2. Averages of first 20 and last 20 trials in the baseline phase (BS) for reaction time, movement time, and step accuracy before applying tDCS

|  | **Anodal tDCS**  (Mean ± SD) | **Sham tDCS**  (Mean ± SD) | **Statistics**  (F, p-value) |
| --- | --- | --- | --- |
| **Reaction time (sec)** |  |  | G: F(1,18) = 0.07, p = 0.79 |
| First 20 BS trials | 0.426 ± 0.074 | 0.437 ± 0.047 | C: F(1,18) = 9.48, p = 0.007 |
| Last 20 BS trials* | 0.403 ± 0.090 | 0.408 ± 0.058 | I: F(1,18) =0.12, p = 0.74 |
| P0 | 0.378 ± 0.084 | 0.410 ± 0.048 |  |
| P3 | 0.371 ± 0.076 | 0.427 ±0.062 |  |
| **Movement time (sec)** |  |  | G: F(1,18) = 0.07, p = 0.79 |
| First 20 BS trials | 0.666 ± 0.086 | 0.638 ± 0.106 | C: F(1,18) = 18.51, p = 0.0004 |
| Last 20 BS trials* | 0.577 ± 0.088 | 0.562 ± 0.104 | I: F(1,18) =0.12, p = 0.74 |
| P0 | 0.562 ± 0.105 | 0.557 ± 0.128 |  |
| P3 | 0.554 ± 0.083 | 0.558 ± 0.123 |  |
| **Step accuracy (cm)** |  |  | G: F(1,18) = 0.07, p = 0.79 |
| First 20 BS trials | 6.150 ± 1.418 | 5.125 ± 0.871 | C: F(1,18) = 10.20, p = 0.005 |
| Last 20 BS trials* | 4.948 ± 1.292 | 4.658 ± 1.303 | I: F(1,18) =0.12, p = 0.74 |
| P0 | 5.127 ± 1.163 | 4.259 ±1.317 |  |
| P3 | 4.918 ± 0.778 | 4.047 ± 1.109 |  |

Note: step accuracy is quantified by the linear distance between the end-point foot position during a forward step and the location of the visual target in the horizontal plane (i.e. X-Y plane). Statistics: Two-way ANOVA mixed model repeated measures was used to compare between Anodal and Sham groups at two time points before brain stimulation: averages of the first 20 trials and the last 20 trials in the baseline phase. G: main effect of group. C: main effect of condition. I: Interaction effect of group by time. *: a significant difference between averages of the first 20 and the last 20 BS trials. P0: 0-min post tDCS. P3: 30-min post tDCS. SD, standard deviation.
